# The Number of Candidate Variants in Exome Sequencing for Mendelian Disease under No Genetic Heterogeneity

**DOI:** 10.1155/2013/179761

**Published:** 2013-05-14

**Authors:** Jo Nishino, Shuhei Mano

**Affiliations:** ^1^Center for Information Biology and DNA Data Bank of Japan, National Institute of Genetics, Research Organization of Information and Systems, 1111 Yata, Mishima, Shizuoka 411-8540, Japan; ^2^Department of Mathematical Analysis and Statistical Inference, The Institute of Statistical Mathematics, Research Organization of Information and Systems, 10-3 Midori-cho, Tachikawa, Tokyo 190-8562, Japan

## Abstract

There has been recent success in identifying disease-causing variants in Mendelian disorders by exome sequencing followed by simple filtering techniques. Studies generally assume complete or high penetrance. However, there are likely many failed and unpublished studies due in part to incomplete penetrance or phenocopy. In this study, the expected number of candidate single-nucleotide variants (SNVs) in exome data for autosomal dominant or recessive Mendelian disorders was investigated under the assumption of “no genetic heterogeneity.” All variants were assumed to be under the “null model,” and sample allele frequencies were modeled using a standard population genetics theory. To investigate the properties of pedigree data, full-sibs were considered in addition to unrelated individuals. In both cases, particularly regarding full-sibs, the number of SNVs remained very high without controls. The high efficacy of controls was also confirmed. When controls were used with a relatively large total sample size (e.g., *N* = 20, 50), filtering incorporating of incomplete penetrance and phenocopy efficiently reduced the number of candidate SNVs. This suggests that filtering is useful when an assumption of no “genetic heterogeneity” is appropriate and could provide general guidelines for sample size determination.

## 1. Introduction

Understanding associations between human genetic variations and phenotypes, including risk of disease, is important for successful realization of personalized medicine. Such variants can be used as biomarkers. Recent advances in high-throughput sequencing technology (“next-generation DNA sequencing” (NGS)) enable exploration of human genetic variations on genome-wide and individual levels. 

The international “1,000-Genome Project,” which uses NGS technology, was launched in 2008. The project aims to create a detailed catalog of human genetic variations by sequencing at least 1,000 individuals [1]. This type of catalog would provide a basis for studies on disease-causing variants or genes. In the last decade, genome-wide association studies (GWAS) using single-nucleotide polymorphism (SNP) genotyping arrays have been successful, although genetic variants identified by GWAS only explain a small proportion of heritability for many complex diseases [2]. A major reason for this limitation is that the “common disease, common variant” hypothesis is a prerequisite for GWAS [2]. The hypothesis that many common diseases are caused by “common variants” (i.e., variants present in more than 1–5% of a population) as detected by SNP genotyping arrays is not likely realistic. Attention has been gradually turned to “rare variants,” which can be detected by NGS technology.

The cost of DNA sequencing is continuously being reduced. However, whole genome sequencing is still too expensive. Recently, sequencing the exome (all protein-coding regions in the genome) has been considered for identifying disease-causing genes or variants. The human exome sequence consists of approximately 30 Mb pairs (nucleotides), corresponding to approximately 1% of the total genome. Thus, exome sequencing is cost effective. Ng et al. [3] provided a proof of concept that exome sequencing can be used to identify disease-causing genes or variants using a simple filtering approach. To date, more than 100 disease-causing genes for Mendelian disorders have been identified using exome sequencing [4].

Analyses of exome data for Mendelian disorders are conducted in a simple, intuitive manner. For example, Ng et al. [3] “reidentified” the MYH3 gene, which is known to cause the rare autosomal dominant disorder Freeman-Sheldon syndrome, as follows: (1) retention of genes in which at least one nonsynonymous single-nucleotide variant (SNV), splice-site variation or indel was present in four unrelated affected individuals and (2) filtering out (removing) variants present in the exomes of eight control individuals or samples from a public database (dbSNP). As an example of using whole genome sequencing for a single patient in a pedigree, Sobreira et al. [5] identified the causative gene of the rare autosomal dominant disease metachondromatosis. In advance linkage analysis using SNP genotyping arrays was conducted, and whole genomes of a single patient and eight unrelated controls were sequenced. The researchers focused on regions with high positive LOD scores and used sequences from the eight controls and dbSNP data as filters to remove variants. They then identified a patient-specific deletion in an exon of PTPN11.

Exome sequencing is an effective method for identifying disease-causing variants in Mendelian disorders. However, there are likely a large number of failed and unpublished studies due to incomplete penetrance, phenocopy, or genotyping error (including sequencing error). Is exome analysis for Mendelian disease actually applicable under assumptions of incomplete penetrance and phenocopy? What is the necessary sample size? To answer such questions, theoretical, simple model studies are suitable. Theoretical research is rarely used for exome analysis in Mendelian disease, even in cases of complete penetrance and no phenocopy. 

**Figure 1:**
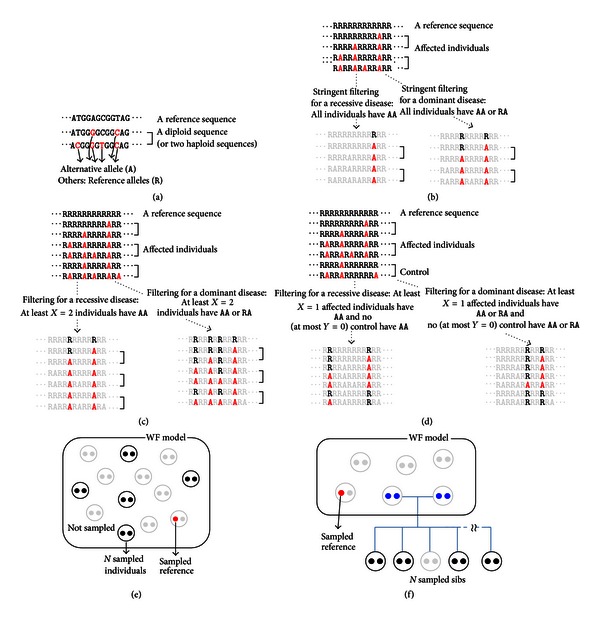
Setting for our study. (a) Alternative (*A*) allele and reference (*R*) allele. (b) Stringent filtering for affected individuals. (c) Filtering incorporating phenocopy. (d) Filtering incorporating incomplete penetrance and phenocopy. (e) Case of unrelated individual. (f) Case of full-sibs.

In exome sequencing, short reads produced by NGS are mapped to the reference sequence, which is the standard human genome sequence, and variants are detected against the reference (Figure 1(a)). Disease-causing variants are searched for based on variants detected in affected individuals. In this study, the number of candidate SNVs for diseases following Mendelian inheritance modes, including autosomal dominant and recessive, was investigated under the assumption of “no genetic heterogeneity” (i.e., no allelic or locus heterogeneity or situations in which a genetic disease is caused by a variant on a gene instead of several variants on one or more genes). It was assumed that allelic types of all variants are independent of the affected status (i.e., all variants are under the “null model”). This is valid because there is only one disease-causing variant. Allelic frequencies in a sample were modeled using a standard population genetics theory. Exome sequences with and without controls were considered, and incomplete penetrance and phenocopy were incorporated as filtering conditions (Figures 1(b), 1(c), and 1(d)). Differences between data from unrelated individuals and pedigrees were also evaluated (Figures 1(e) and 1(f)). Public databases (e.g., dbSNP or 1,000 Genome Project database), which can include errors and generally do not provide phenotype information, are often used to filter out SNVs in exome analysis, but were not considered in this study. Zhi and Chen [6] modeled an analysis of exome sequencing. The authors investigated the power of various conditions, including the number of mutations identified after filtering (corresponding to the number of SNVs after filtering in this study), inheritance modes of disease (i.e., autosomal dominant and recessive), locus heterogeneity, gene length, sample size, and others. Common or low quality variants were filtered out in advance and disease-causing genes were explored under genetic heterogeneity. The authors treated the number of SNVs after filtering as a known constant. In contrast, we directly filtered disease-causing variants according to modes of inheritance under the assumption of “no genetic heterogeneity” and evaluated the number of candidate SNVs after filtering. In addition, although the term “SNV” means “single-nucleotide variant” as shown in Figure 1(a), it can be interpreted simply as a “variant,” including “splice-site variant” or “indel.” The term “SNV” is used in this study because there are fewer splice-site variants or indels than SNVs in exome sequences [3].

## 2. Method

There are roughly 20,000 SNVs in a single human exome [3]. That is, diploid exome sequences (two haploid exome sequences) have different allelic types (alternative types, *A*) from haploid reference sequences (reference types, *R*) at ~20,000 DNA sites (Figure 1(a)). According to the population genetics theory described below, the expected number of SNVs with *i* mutant and *n* − *i* ancestral alleles in *n* haploid sequences randomly sampled from a population can be obtained using a simple formula. In Section 2.1, we used this formula to derive an expression for the expected number of SNVs with *n*_*A*_ alternative and  *n* − *n*_*A*_ reference alleles in *n* haploid sequences randomly sampled from a population. In Section 2.2, exome sequences of *N* unrelated affected individuals (Figure 1(e)) were considered, and the expected number of SNVs for individuals with genotypes *RR*, *RA*, and *AA* (*n*_*RR*_, *n*_*RA*_ and *n*_*AA*_, resp.) was obtained. This enabled calculation of the expected number of SNVs after filtering, as illustrated in Figures 1(b) and 1(c). In Section 2.3, a case with additional controls was considered (Figure 1(d)). In Section 2.4, we considered data from full-sibs with and without controls in a nuclear family to investigate the properties of the expected number of SNVs using exome sequences from a pedigree (Figure 1(f)).

### 2.1. Site Frequency Spectrum of the Alternative Allele

We considered *n* haploid sequences randomly sampled from a population under the Wright-Fisher diffusion model. The infinite-site model of neutral mutations was assumed. We denoted the diploid population size and mutation rate per haploid sequence per generation by *PopSize* and *μ*, respectively. *M*_*i*_ indicates the number of SNVs with *i* mutant (derived) and *n* − *i* ancestral alleles in *n* haploid sequences. *M*_*i*_ is the “site frequency spectrum” of the mutant (derived) allele in a sample. According to Fu [7], the expectation of *M*_*i*_ is the result of

(1) $$\begin{array}{c} E\left[M_{i}\right]=\frac{\theta }{i},\;1\leq i\leq n-1, \end{array}$$

where *θ* = 4 × *PopSize* × *μ*. This simple formula does not include the sample size *n*. As described in the following section, the point estimate of *θ* for the human exome is ~13,333. For example, when considering four haploid exomes (equivalent to two unrelated diploid exomes), the number of SNVs with one, two, and three mutant alleles is expected to be 13,333, 6,666.50, and 4,444.33, respectively. 

However, in practice it is often not known if the DNA type at a segregating site is mutant or ancestral. In exome analysis, DNA types are generally expressed as “reference (*R*)” or “alternative (*A*)” because variants in exome sequences are detected based on comparison with a reference genome sequence (Figure 1(a)). This study was also carried out in terms of “Reference type (*R*)” or “Alternative type (*A*)”. Thus, as a first step, we defined *M*_*n*_*A*__′ in place of *M*_*i*_ to derive the expression *E*[*M*_*n*_*A*__′].

In addition to *n* haploid sequences, we considered that a reference sequence was also randomly sampled from a population (*n* + 1 sequences). We defined *M*_*n*_*A*__′ as the number of SNVs with *n*_*A*_ alternative and *n* − *n*_*A*_ reference alleles in the *n* haploid sequences. In a segregating site in *n* + 1 sequences, reference DNA is either mutant or ancestral. The expected number of SNVs in which reference DNA is mutant and *n*_*R*_  reference alleles in the *n* haploid sequences is derived by the product of the expected number of SNVs with *n*_*R*_ + 1 mutant alleles in *n* + 1 sequences, *θ*/(*n*_*R*_ + 1) based on (1), and the probability that a mutant allele is chosen as a reference from *n* + 1 alleles with *n*_*R*_ + 1 mutant alleles, (*n*_*R*_ + 1)/(*n* + 1). This is represented as

(2) $$\begin{array}{c} \frac{\theta }{n_{R}+1}\frac{n_{R}+1}{n+1}=\frac{\theta }{n+1}. \end{array}$$

Similarly, the expected number of SNVs in which reference DNA is ancestral and *n*_*R*_  reference alleles in *n* haploid sequences was obtained. The expectation is represented as the product of the expected number of SNVs with (*n* + 1) − (*n*_*R*_ + 1) = (*n* − *n*_*R*_) mutant alleles in (*n* + 1) sequences, *θ*/(*n* − *n*_*R*_) based on (1), and the probability that a mutant allele is chosen as a reference from *n* + 1 alleles with *n*_*R*_ + 1 mutant alleles, (*n*_*R*_ + 1)/(*n* + 1). The resulting equation is

(3) $$\begin{array}{c} \frac{\theta }{n-n_{R}}\frac{n_{R}+1}{n+1}. \end{array}$$

The expectation of *M*_*n*_*A*__′, *E*[*M*_*n*_*A*__′], is equal to the sum of (2) and (3), resulting in

(4) E[MnA′]=θn+1+θn−nRnR+1n+1=θn−nR=θnA, 1≤nA≤n.

The formula does not include sample size. Interestingly, this result is obtained by (1), assuming that the alternative alleles are a mutant. Note that *n*_*A*_ can be equal to *n* at most in (4) (*n* alleles are all alternatives at a particular DNA site).

### 2.2. Unrelated *N* Affected Individuals

Next, consider exome sequences of unrelated *N* affected individuals under the Wright-Fisher diffusion model (Figure 1(e)). The infinite-site model of neutral mutations was assumed again. Assuming that *N* diploid exome sequences and a reference sequence are “randomly sampled” from the population, we obtained the expected number of SNVs in which the number of individuals with genotypes *RR*, *RA*, and *AA* is *n*_*RR*_,  *n*_*RA*_, and *n*_*AA*_, respectively. Here, “randomly sampled” means that *N* diploid exome sequences and a reference sequence are “randomly sampled” (*N* + 1 times), which is equivalent to 2*N* + 1 haploid exome sequences that are “randomly sampled” (2*N* + 1 times), followed by one sequence chosen as a reference from the 2*N* + 1 sequences. The remaining 2*N* sequences are randomly joined to form *N* diploids. The latter is used for illustrative purposes.

Conditions of the variables were collected. As in Section 2.1 *n*_*R*_ and *n*_*A*_ denote the number of reference and alternative alleles in a site, respectively. One has

(5a) $$\begin{array}{c} n_{R},n_{A},n_{RR},n_{RA},n_{A}\;\in \;\text{nonnegative}\;\text{integers}, \end{array}$$

(5b) 2N=nR+nA,                        (5b.1)N=nRR+nRA+nAA,      (5b.2)nR=2nRR+nRA,                  (5b.3)nA=2nAA+nRA.                 (5b.4)

Note that given *n*_*A*_ or *n*_*R*_ (and constant *N*), there is only one independent variable among *n*_*RR*_,  *n*_*RA*_ and *n*_*AA*_. For example, if *n*_*A*_, and *n*_*AA*_ are fixed, the other two variables, *n*_*RR*_ and *n*_*RA*_, are automatically determined.

Let *K*(*N*, *n*_*R*_, *n*_*A*_, *n*_*RR*_, *n*_*RA*_, *n*_*AA*_)  be  the number of SNVs in which the number of reference and alternative alleles is *n*_*R*_ and *n*_*A*_, respectively, and the number of individuals with genotypes *RR*, *RA*, and *AA* is *n*_*RR*_, *n*_*RA*_, and *n*_*AA*_, respectively, in total *N* individuals. The expected number of SNVs, *E*[*K*(*N*, *n*_*R*_, *n*_*A*_, *n*_*RR*_, *n*_*RA*_, *n*_*AA*_)], is defined only when all conditions of (5a) and (5b) are met. First, we considered 2*N* haploid exome sequences and a reference to be “randomly sampled” (2*N* + 1 times). The number of SNVs with *n*_*A*_ alternative and *n* − *n*_*A*_ reference alleles in the 2*N* + 1 haploid samples can be readily obtained by (4). The probability that the genotype configuration (*n*_*RR*_, *n*_*RA*_, *n*_*AA*_) was determined given that a DNA site has *n*_*A*_ alternative alleles was denoted as Prob(*n*_*RR*_, *n*_*RA*_, *n*_*AA*_ | *n*_*A*_). The number of distinct permutations of 2*N* is given by (2*N*)!/(*n*_*R*_!*n*_*A*_!). How many permutations result in the genotype configuration (*n*_*RR*_, *n*_*RA*_, *n*_*AA*_)? The number of ways to determine the genotype of each individual in distinct *N* individuals and generate a genotype configuration of (*n*_*RR*_, *n*_*RA*_, *n*_*AA*_) is equal to *N*!/(*n*_*RR*_!*n*_*RA*_!*n*_*AA*_!). The genotype *RA* can be generated from the two runs, *RA* and *AR*. Therefore, the number of permutations used to generate the genotype configuration (*n*_*RR*_, *n*_*RA*_, *n*_*AA*_) is derived from (2^*n*_*RA*_^ *N*!)/(*n*_*RR*_!*n*_*RA*_!*n*_*AA*_!) and Prob(*n*_*RR*_, *n*_*RA*_, *n*_*AA*_ | *n*_*A*_) = (2^*n*_*RA*_^ *N*!)/(*n*_*RR*_!*n*_*RA*_!*n*_*AA*_!)×(*n*_*R*_!*n*_*A*_!)/(2*N*)!. The expression of Prob(*n*_*RR*_, *n*_*RA*_, *n*_*AA*_ | *n*_*A*_) was shown elsewhere and used to perform the exact test of Hardy-Weinberg equilibrium [8]. Let us give a proof of the following proposition.

**Proposition 1**

*E*[*K*(*N*, *n*_*R*_, *n*_*A*_, *n*_*RR*_, *n*_*RA*_, *n*_*AA*_)] = *E*[*M*_*n*_*A*__′] × Prob (*n*_*RR*_, *n*_*RA*_, *n*_*AA*_ | *n*_*A*_).

**Proof**

*E*[*K*] = *E*_diff_[*E*_samp_[*K* | *M*_*n*_*A*__′]], where *E*_diff_ is the expectation with respect to the diffusion model and *E*_samp_ is the expectation with respect to the binomial sampling. Binomial sampling is *M*_*n*_*A*__′-times Bernoulli trial, addressing whether a site indicates genotype counts of (*n*_*RR*_, *n*_*RA*_, *n*_*AA*_). Probability of the Bernoulli trial is Prob(*n*_*RR*_, *n*_*RA*_, *n*_*AA*_ | *n*_*A*_). Therefore, *E*_diff_[*E*_samp_[*K* | *M*_*n*_*A*__′]] = *E*_diff_[*M*_*n*_*A*__′Prob(*n*_*RR*_, *n*_*RA*_, *n*_*AA*_ | *n*_*A*_)] = *E*_diff_[*M*_*n*_*A*__′]Prob(*n*_*RR*_, *n*_*RA*_, *n*_*AA*_ | *n*_*A*_). *E*_diff_[*M*_*n*_*A*__′] is represented by (4) and the proposition follows.

Then, we have

(6) E[K(N,nR,nA,nRR,nRA,nAA)]  =E[MnA′]×Prob(nRR,nRA,nAA ∣ nA)  =θnA×2nRAN!nRR!nRA!nAA!nR!nA!(2N)!.

Here, *E*[*K*(*N*, *n*_*R*_, *n*_*A*_, *n*_*RR*_, *n*_*RA*_, *n*_*AA*_)] is not defined if (5a) and (5b) are not satisfied. For example, in the case of *N* = 2 (4 haploid sequences), the expected number of SNVs for (*N*, *n*_*R*_, *n*_*A*_, *n*_*RR*_, *n*_*RA*_, *n*_*AA*_) = (2,3, 1,1, 1,0), (2,2, 2,0, 2,0), (2,2, 2,1, 0,1), (2,1, 3,0, 1,1), (2,0, 4,0, 0,2) satisfying (5a) and (5b) is *θ*, *θ*/3, *θ*/6, *θ*/3, and *θ*/4, respectively. If we use 13,333 as human exome *θ*, *E*[*K*(*N*, *n*_*R*_, *n*_*A*_, *n*_*RR*_, *n*_*RA*_, *n*_*AA*_)] is 13,333, 4,444.33, 2,222.17, 4,444.33, and 3,333.25, respectively. If both individuals are affected by a certain recessive disease with the genotype *AA* at a causal DNA site, we can use a filter to retain variants in which both individuals have the genotype *AA*. The expected number of SNVs after filtering is *E*[*K*(2,0, 4,0, 0,2)] = 3,333.25. Similarly, when both individuals are affected by a dominant disease with genotypes *AA* or *RA* at a causal DNA site, the expected number of SNVs after filtering is *E*[*K*(2,2, 2,0, 2,0)] + *E*[*K*(2,1, 3,0, 1,1)] + *E*[*K*(2,0, 4,0, 0,2)] = 4,444.33 + 4,444.33 + 3,333.25 = 12,221.91. In this way, by summing *E*[*K*(*N*, *n*_*R*_, *n*_*A*_, *n*_*RR*_, *n*_*RA*_, *n*_*AA*_)] for all sets of (*N*, *n*_*R*_, *n*_*A*_, *n*_*RR*_, *n*_*RA*_, *n*_*AA*_) that satisfy (5a) and (5b) and including a filtering condition, the expected number of SNVs after filtering can be calculated.

In some cases, factors such as reduced penetrance, phenocopy (including misdiagnosis), or genotyping errors should be taken into account. So, consider filtering to retain only SNVs in which at least *X*(≥*X*) of *N* affected individuals have *AA* in cases of recessive disease or *AA* or *RA* in cases of dominant disease (Figure 1(c)). At the disease-causing variant site, this allows the phenocopy (or genotyping error) from genotype *RR* or *RA* to *AA* in cases of recessive disease or from genotype *RR* to *AA* or *RA* in cases of dominant disease. The following are detailed methods of calculating the expected number of SNVs after filtering.

As noted, given *n*_*A*_ or *n*_*R*_ (and constant *N*), there is only one independent variable in the conditions of (5b). In case of recessive disease, we can express *K*(*N*, *n*_*R*_, *n*_*A*_, *n*_*RR*_, *n*_*RA*_, *n*_*AA*_) as a function of *N*, *n*_*A*_, *n*_*AA*_ using (5b), denoted by *E*[*K*(*N*,*n*_*A*_, *n*_*AA*_)]. Specifically, this can be expressed as

(7) E[K(N,nA,nAA)]  =θnA2(nA−2nAA)N!(N−nA+nAA)!(nA−2nAA)!nAA!   ×(2N−nA)!nA!(2N)!.

*E*[*K*(*N*,*n*_*A*_, *n*_*AA*_)] is not defined if (5a) is not satisfied. After filtering, the expected number of SNVs in which at least *X*(≥*X*) of *N* affected individuals have *AA* is calculated by

(8) $$\begin{array}{c} \sum_{n_{A},\;X\leq n_{AA}}E\left[K\left(N{,n}_{A},n_{AA}\right)\right]. \end{array}$$

In cases of dominant disease, denoting *n*_*AA*+*RA*_ as the number of individuals with genotypes *AA* or *RA* (*n*_*AA*+*RA*_ = *n*_*AA*_ + *n*_*RA*_) can be expressed as *E*[*K*(*N*, *n*_*R*_, *n*_*A*_, *n*_*RR*_, *n*_*RA*_, *n*_*AA*_)] as a function of *N*, *n*_*A*_, *n*_*AA*+*RA*_ using (5b), denoted by *E*[*K*(*N*,*n*_*A*_, *n*_*AA*+*RA*_)]. This results in

(9) E[K(N,nA,nAA+RA)]  =θnA2(2nAA+RA−nA)N!(N−nAA+RA)!(2nAA+RA−nA)!(nA−nAA+RA)!   ×(2N−nA)!nA!(2N)!.

*E*[*K*(*N*,*n*_*A*_, *n*_*AA*+*RA*_)] is not defined if (5a) is not satisfied. After filtering, the expected number of SNVs in which at least QUOTE *X*(≥*X*) of *N* affected individuals have *AA* or *RA* is calculated by

(10) $$\begin{array}{c} \sum_{n_{A},\;X\leq n_{AA+RA}}E\left[K\left(N{,n}_{A},n_{AA+RA}\right)\right]. \end{array}$$

### 2.3. Unrelated *N*_*a*_ Affected Individuals with *N*_*c*_ Controls

Consider exome sequences of unrelated *N* individuals consisting of *N*_*a*_ affected individuals and *N*_*c*_ controls. In cases of recessive disease, we considered a filter to retain only SNVs in which at least QUOTE *X*(≥*X*) of *N*_*a*_ affected and at most QUOTE *Y*(≤*Y*) of *N*_*c*_ control individuals have *AA* (Figure 1(d), left). This allows the phenocopy (or genotyping error) from genotype *RR* or *RA* to *AA* and/or the reduced penetrance of *AA* at a disease-causing variant site. Similarly, in cases of dominant disease, we considered a filter to retain only SNVs in which at least QUOTE *X*(≥*X*) of *N*_*a*_ affected and at most QUOTE *Y*(≤*Y*) of *N*_*c*_ control individuals have *AA* or *RA* (Figure 1(d), right).

First we did not distinguish affected individuals from controls in total *N* individuals. The expected number of SNVs in which the number of alternative alleles is *n*_*A*_ and the number of individuals with genotype *AA* is *n*_*AA*_ is still given by (7). Next we assumed that *N*_*a*_ affected individuals and *N*_*c*_ controls were randomly selected from *N* individuals. Considering recessive diseases, for a given *n*_*AA*_, the number of individuals with genotypes *AA*, *n*_*AA*(*a*)_, in *N*_*a*_ affected individuals follows a hypergeometric distribution. As a result, the expected number of SNVs, *E*[*K*_2_(*N*, *N*_*a*_, *n*_*A*_, *n*_*AA*_, *n*_*AA*(*a*)_)], in which the number of alternative alleles is *n*_*A*_ and the number of individuals with genotype *AA* is *n*_*AA*(*a*)_ in *N*_*a*_ affected individuals is represented as

(11)   E[K2(N,Na,nA,nAA,nAA(a))]  =Prob(nAA(a) ∣ nA,nAA)×E[K(N,nA,nAA)]  =(nAAnAA(a))×(N−nAANa−nAA(a))(NNa)E[K(N,nA,nAA)].

After filtering, the expected number of SNVs in which at least *X*(≥*X*) of *N*_*a*_ affected individuals and at most *Y*(≤*Y*) of *N*_*c*_ control individuals have the *AA* genotype is obtained by summing *E*[*K*_2_]:

(12) $$\begin{array}{c} \sum_{n_{A},\;n_{AA},\;n_{AA(a)}}E\left[K_{2}\left(N,N_{a},n_{A},n_{AA},n_{AA(a)}\right)\right], \end{array}$$

where the sum of *n*_*AA*(*a*)_ is over the value satisfying the filtering condition, {*n*_*AA*(*a*)_ : *X* ≤ *n*_*AA*(*a*)_∧*n*_*AA*(*c*)_ = (*n*_*AA*_ − *n*_*AA*(*a*)_) ≤ *Y*}. *n*_*AA*(*c*)_ denotes the number of individuals with *AA* genotypes in the *N*_*c*_ controls.

Similarly, considering dominant diseases the expected number of SNVs in which the number of alternative alleles is *n*_*A*_ and the number of individuals with genotypes *AA* or *RA* (*n*_*AA*+*RA*(*a*)_) in *N*_*a*_ affected individuals is represented by

(13) E[K2(N,Na,nA,nAA+RA,nAA+RA(a))] =Prob(nAA+RA(a) ∣ nA,nAA+RA)×E[K(N,nA,nAA+RA)] =(nAA+RAnAA+RA(a))×(N−nAA+RANa−nAA+RA(a))(NNa)E[K(N,nA,nAA+RA)].

After filtering, the expected number of SNVs in which at least *X*(≥*X*) of *N*_*a*_ affected individuals and at most *Y*(≤*Y*) of *N*_*c*_ control individuals have the *AA* or *RA* genotypes is obtained by summing *E*[*K*_2_]:

(14) $$\begin{array}{c} \sum_{n_{A},\;n_{AA},\;n_{AA+R(a)}}E\left[K_{2}\left(N,N_{a},n_{A},n_{AA+RA},n_{AA+RA(a)}\right)\right], \end{array}$$

where the sum of *n*_*AA*+*RA*(*a*)_ is over the value satisfying the filtering condition, {*n*_*AA*+*RA*(*a*)_ : *X* ≤ *n*_*AA*+*RA*(*a*)_∧*n*_*AA*+*RA*(*c*)_ = (*n*_*AA*+*RA*_ − *n*_*AA*+*RA*(*a*)_) ≤ *Y*}. Here, *n*_*AA*+*RA*(*c*)_ denotes the number of individuals with *AA* or *RA* genotypes in *N*_*c*_ controls.

### 2.4. *N* Full-Sibs with and without Controls

To investigate the properties of the number of SNVs using exomes from a pedigree, we considered *N* full-sibs with and without controls in a nuclear family (Figure 1(f)). Assumptions were that four haploid exome sequences of both parents and a reference sequence were randomly sampled from a population under the Wright-Fisher diffusion model. The infinite-site model of neutral mutations was also assumed.

The expected number of SNVs with a particular genotype configuration from both parents was obtained by (6). Otherwise using formula (4), the expected number was obtained as follows: the expected number of SNVs with both parents genotypes *RR* × *RA*, *RA* × *AA*, and *AA* × *AA* is readily obtained by substituting *n*_*A*_ = 1, 3 and 4 into (4) to be *θ*, *θ*/3 and *θ*/4, respectively. Here, *RR* × *RA* indicates that the genotype of one parent is *RR* and that of the other is *RA*, and so on. Although the expected number of SNVs in which *n*_*A*_ = 2 in four haploid sequences is *θ*/2 by substituting *n*_*A*_ = 2 into (4), SNVs likely result in two genotype configurations, *RR* × *AA* and *RA* × *RA*. Considering random combinations of {*R*, *R*, *A*, *A*}, the expected number of SNVs with *RR* × *AA* and *RA* × *RA* is represented by $\theta /2\times 2\times \left(\begin{array}{c} 2\\ 2 \end{array}\right)/\left(\begin{array}{c} 4\\ 2 \end{array}\right)=1/6\theta$, $\theta /2\times 2/\left(\begin{array}{c} 4\\ 2 \end{array}\right)=1/3\theta$. Given the genotype configuration of both parents, the number of sibs with genotypes *RR*, *RA*, and *AA* follows a polynomial distribution. For possible genotype configurations of both parents, Table 1 shows the expected number of SNVs and probabilities that a sib with a particular genotype would be born (i.e., parameters of a polynomial distribution). 

The expected number, *E*[*K*_sib_(*n*_*RR*_, *n*_*RA*_, *n*_*AA*_)], of SNVs in which the number of sibs with genotypes *RR*, *RA*, and *AA* is *n*_*RR*_, *n*_*RA*_, and *n*_*AA*_, respectively, is represented as

(15) E[Ksib(nRR,nRA,nAA)]  =E[KRR×RA]×Prob(nRR,nRA,nAA ∣ RR×RA)+⋯   +E[KAA×AA]×Prob(nRR,nRA,nAA ∣ AA×AA)  =θ×N!nRR!nRA!nAA!×12nRR12nRA0nAA   +16θ×N!nRR!nRA!nAA!×0nRR1nRA0nAA   +13θ×N!nRR!nRA!nAA!×14nRR12nRA14nAA   +13θ×N!nRR!nRA!nAA!×0nRR12nRA12nAA   +14θ×N!nRR!nRA!nAA!×0nRR0nRA1nAA,

where *n*_*RR*_ + *n*_*RA*_ + *n*_*AA*_ = *N*, 0^0^ = 1 and 0^1^ = 0^2^ = ⋯ = 0. Being simplified, this is shown as

(16) E[Ksib(nRR,nRA,nAA)]  =N!nRR!nRA!nAA!θ   ×  {12nRR+nRA0nAA+160nRR+nAA+13122nRR+nRA+2nAA     +130nRR12nRA+nAA+140nRR+nRA}.

Here, *n*_*RR*_,  *n*_*RA*_, *n*_*AA*_∈ nonnegative integers and *N* = *n*_*RR*_ + *n*_*RA*_ + *n*_*AA*_. Using (16), the expected number of SNVs after filtering is calculated as shown. This calculation is easier than that in unrelated individuals. In recessive diseases, the expected number of SNVs in which at least *X*(≥*X*) of *N* affected individuals have *AA* after filtering is calculated as

(17) $$\begin{array}{c} \sum E\left[K_{\text{sib}}\left(n_{RR},n_{RA},n_{AA}\right)\right], \end{array}$$

where the summation is over (*n*_*RR*_, *n*_*RA*_, *n*_*AA*_), satisfying the filter condition {(*n*_*RR*_, *n*_*RA*_, *n*_*AA*_) : *X* ≤ *n*_*AA*_}. Similarly, in cases of dominant disease, the expected number of SNVs in which at least QUOTE *X*(≥*X*) of *N* affected individuals have an *AA* genotype after filtering is calculated using (17), where if *n*_*AA*+*RA*_ = *n*_*AA*_ + *n*_*RA*_, the summation is over (*n*_*RR*_, *n*_*RA*_, *n*_*AA*_), satisfying the filter condition {(*n*_*RR*_, *n*_*RA*_, *n*_*AA*_) : *X* ≤ *n*_*AA*+*RA*_}.

We considered *N*_*a*_ affected sibs with *N*_*c*_ control sibs. Given genotype configurations of both parents at a site, the number of *N*_*a*_ and *N*_*c*_ sibs with genotypes *RR*, *RA*, and *AA* at the site follows independent polynomial distribution. *n*_*RR*(*a*)_, *n*_*RA*(*a*)_, and *n*_*AA*(*a*)_ were the number of *RR*, *RA*, and *RA*, respectively, in *N*_*a*_ affected sibs, and *n*_*RR*(*c*)_, *n*_*RA*(*c*)_, and *n*_*AA*(*c*)_ were the number of *RR*, *RA* and *AA*, respectively, in *N*_*c*_ control sibs. The expected number, *E*[*K*_sib2_(*n*_*RR*(*a*)_, *n*_*RA*(*a*)_, *n*_*AA*(*a*)_, *n*_*RR*(*c*)_, *n*_*RA*(*c*)_, *n*_*AA*(*c*)_)], of SNVs with the genotype configuration of sibs (*n*_*RR*(*a*)_, *n*_*RA*(*a*)_, *n*_*AA*(*a*)_, *n*_*RR*(*c*)_, *n*_*RA*(*c*)_, *n*_*AA*(*c*)_) is represented as

(18) E[Ksib2(nRR(a),nRA(a),nAA(a),nRR(c),nRA(c),nAA(c))] =θ×N(a)!nRR(a)!nRA(a)!nAA(a)!N(c)!nRR(c)!nRA(c)!nAA(c)!  ×{12nRR+nRA0nAA+160nRR+nAA+13122nRR+nRA+2nAA    +130nRR12nRA+nAA+140nRR+nRA},

where *n*_*RR*_ = *n*_*RR*(*a*)_ + *n*_*RR*(*c*)_; *n*_*RA*_ = *n*_*RA*(*a*)_ + *n*_*RA*(*c*)_; *n*_*AA*_ = *n*_*AA*(*a*)_ + *n*_*AA*(*c*)_; *n*_*RR*(a)_, *n*_*RA*(*a*)_, *n*_*AA*(a)_, *n*_*RR*(c)_, *n*_*RA*(*c*)_, *n*_*AA*(c)_∈ nonnegative integers; *N*_(*a*)_ = *n*_*RR*(a)_ + *n*_*RA*(a)_ + *n*_*AA*(a)_ and *N*_(*c*)_ = *n*_*RR*(c)_ + *n*_*RA*(c)_ + *n*_*AA*(c)_. The expected number of SNVs after filtering is calculated as shown just below. In cases of recessive disease, the expected number of SNVs in which at least *X*(≥*X*) of *N*_*a*_ affected and at most QUOTE *Y*(≤*Y*) of *N*_*c*_ control individuals have the genotype *AA* after filtering is obtained by

(19) $$\begin{array}{c} \sum E\left[K_{\text{sib}2}\left(n_{RR(a)},n_{RA(a)},n_{AA(a)},n_{RR(c)},n_{RA(c)},n_{AA(c)}\right)\right], \end{array}$$

where the summation is over (*n*_*RR*(*a*)_, *n*_*RA*(*a*)_, *n*_*AA*(*a*)_, *n*_*RR*(*c*)_, *n*_*RA*(*c*)_, *n*_*AA*(*c*)_), satisfying the filter condition {*n*_*AA*(*a*)_ : *X* ≤ *n*_*AA*(*a*)_∧*n*_*AA*(*c*)_ ≤ *Y*}. Similarly, in cases of dominant disease, the expected number of SNVs in which at least QUOTE *X*(≥*X*) of *N*_*a*_ affected and at most QUOTE *Y*(≤*Y*) of *N*_*c*_ control individuals have *AA* or *RA* after filtering is calculated using (18), where if *n*_*A*+*RA*(*a*)_ = *n*_*AA*(*a*)_ + *n*_*RA*(*a*)_ and *n*_*AA*+*RA*(*c*)_ = *n*_*AA*(*c*)_ + *n*_*RA*(*c*)_, the summation is over (*n*_*RR*(*a*)_, *n*_*RA*(*a*)_, *n*_*AA*(*a*)_, *n*_*RR*(*c*)_, *n*_*RA*(*c*)_, *n*_*AA*(*c*)_), satisfying the filter condition {(*n*_*RR*_, *n*_*RA*_, *n*_*AA*_) : *X* ≤ *n*_*AA*+*RA*(*a*)_∧*n*_*AA*+*RA*(*c*)_ ≤ *Y*}.

## 3. Results and Discussion

### 3.1. An Estimator of *θ* for Human Exome

According to Table 2 in Ng et al. [3], there are roughly 20,000 SNVs in a single human exome, including synonymous and non-synonymous variants. All results in this study are based on the estimate $\hat{\theta }=$ 13,333, which was obtained based on 20,000 SNVs per individual as follows: the expected number of SNVs detected in one human is represented as *E*[*M*_*n*_*A*_=1_′] + *E*[*M*_*n*_*A*_=2_′] = 3*θ*/2 using (4), with possible *n* = 2 values of *n*_*A*_ ∈ {1,2}. If the observed number of SNVs detected in one human is 20,000, then 3*θ*/2 = 20,000 is used to obtain $\hat{\theta }=$ 13,333. Note that the number of SNVs per single human exome (20,000) varies between races and is based on different methods of exome capture, mapping to a reference genome, genotype calling algorithms, or by definition of an exome. The results of this study also varied slightly based on the $\hat{\theta }$ estimators used.

### 3.2. Unrelated Individuals without Controls in Dominant Disease

**Figure 2:**
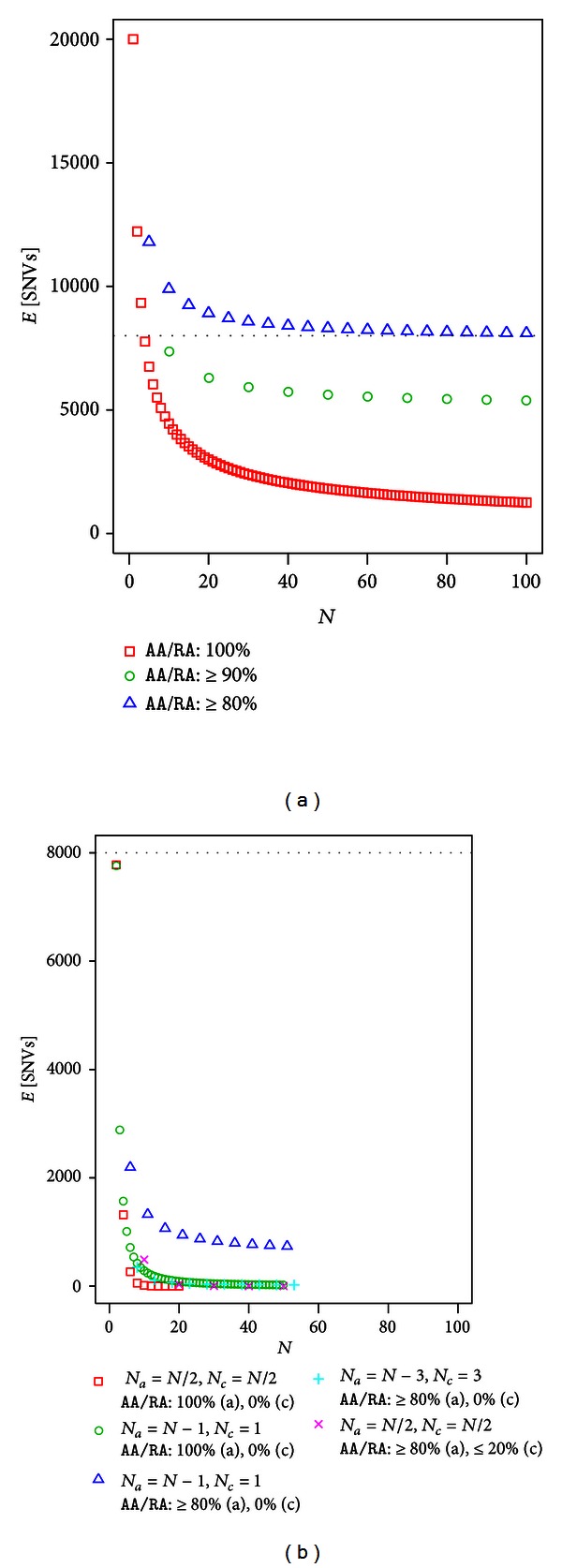
The expected number of SNVs after filtering in dominant disease using unrelated individuals (a) without controls (b) with controls. For example, cross marks represent the expected number of SNVs in which ≥80% individuals have the genotype *AA*/*RA* of *N*_*a*_ = *N*/2 affected individuals and ≤20% individuals have the genotype *AA*/*RA* of *N*_*c*_ = *N*/2 controls.

The expected number of SNVs after filtering in cases of dominant disease and unrelated individuals without controls is plotted in Figure 2(a). Several values used in Figure 2 are listed in Table 2. When a stringent filter (i.e., set to retain only SNVs in which 100% of individuals sampled have the genotype *AA*/*RA*) was used, the number of SNVs appeared to decay exponentially with sample size *N*. However, the decrease in the number of SNVs was slower as *N* increased. As shown in Table 2, the expected number of SNVs for *N* = 1, 2, 3, 4, 50, and 51 were 19999.50, 12221.92 (61.11%), 9333.10 (76.36%), 7761.71 (83.16%), 1808.56, and 1789.36 (98.94%), respectively, with ratios of the expected SNVs for *N* to those for *N* − 1 shown in parentheses. The first few individuals were highly effective in removing SNVs, but additional individuals were not. This was obvious when nonstringent filters (i.e., remaining SNVs in which at least 90% or 80% of individuals have the genotype *AA*/*RA*) were used. In those cases, certain asymptotic values likely exist. For example, ≥90% of the filtered expected number of SNVs was 5540.39 for *N* = 50, but only 5306.36 for *N* = 100. From the perspective of identifying disease-causing variants, it is clear that nonstringent filters that take phenocopy into account do not work well even if the sample size is very large. However, using stringent filters, the expected number of SNVs remains high even if the sample size is large (1249.75 SNVs for *N* = 100). This shows that it is generally difficult to identify a disease-causing variant by filtering without a control.

### 3.3. Unrelated Individuals with Controls in Dominant Disease

As shown in Figure 2(b), filtering with controls is highly effective in removing SNVs. When half of the samples were controls and a stringent filter was used, the expected number of SNVs was less than one at *N* = 14 and 0.001 at *N* = 20. Even with a single control, the situation changed drastically compared to cases without controls. For example, for *N* = 10, the expected number of SNVs was 4450.2 without a control, which dropped to 284.27 with one control. Using nonstringent filters that take phenocopy into account (i.e., remaining SNVs in which 80% of affected individuals have the genotype *AA*/*RA*), an asymptotic value of approximately 700 may occur with one control, but filtering efficiency is improved if the number of controls totals 3 (21.74 SNVs for *N* = 53). In addition to phenocopy, filters that take reduced penetrance into account also work reasonably well if half of the exome samples (*N*/2) are controls. For example, the expected number of SNVs in which 80% of affected individuals and 20% of controls have the genotype *AA*/*RA* was 28.51 and 0.02 for *N* = 20 and 50, respectively.

### 3.4. Unrelated Individuals and Recessive Disease

**Figure 3:**
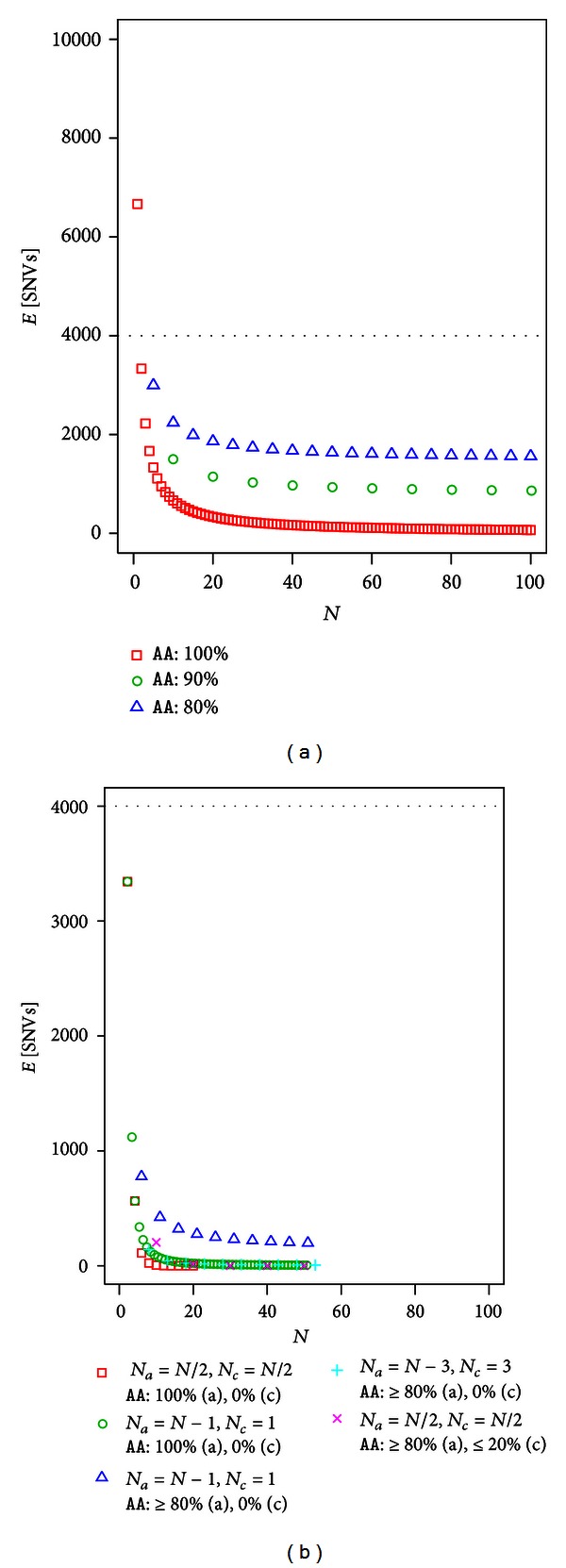
The expected number of SNVs after filtering in recessive disease using unrelated individuals (a) without control using and (b) with controls.

The number of SNVs after filtering in recessive disease shows a similar tendency to SNVs in dominant disease, as shown in Figures 3(a) and 3(b).  Table 3 lists some of the values used in Figure 3. Without controls, filtering does not work well, particularly when phenocopy is taken into account. With controls, filtering efficiency is highly improved even when phenocopy and reduced penetrance are considered. However, filtering efficiency for recessive disease is at most ten times higher compared to dominant disease. For example, stringent filtering of *N* = 100 without a control resulted in an expected number of SNVs of 1249.75 for dominant disease, but only 66.67 for recessive disease. Using stringent filtering, the expected number of SNVs for recessive disease was

(20) $$\begin{array}{c} \frac{\theta }{2N}, \end{array}$$

which is derived from (7) or directly from (4) by substituting *n*_*A*_ = 2*N*. In contrast, the expected number of SNVs for dominant disease is represented as

(21) ∑i=N2Nθi2(2N−i)(2N2N−i)(2N2N−i),

which is derived from (9) and (10).

### 3.5. Full-Sibs with and without Controls

**Figure 4:**
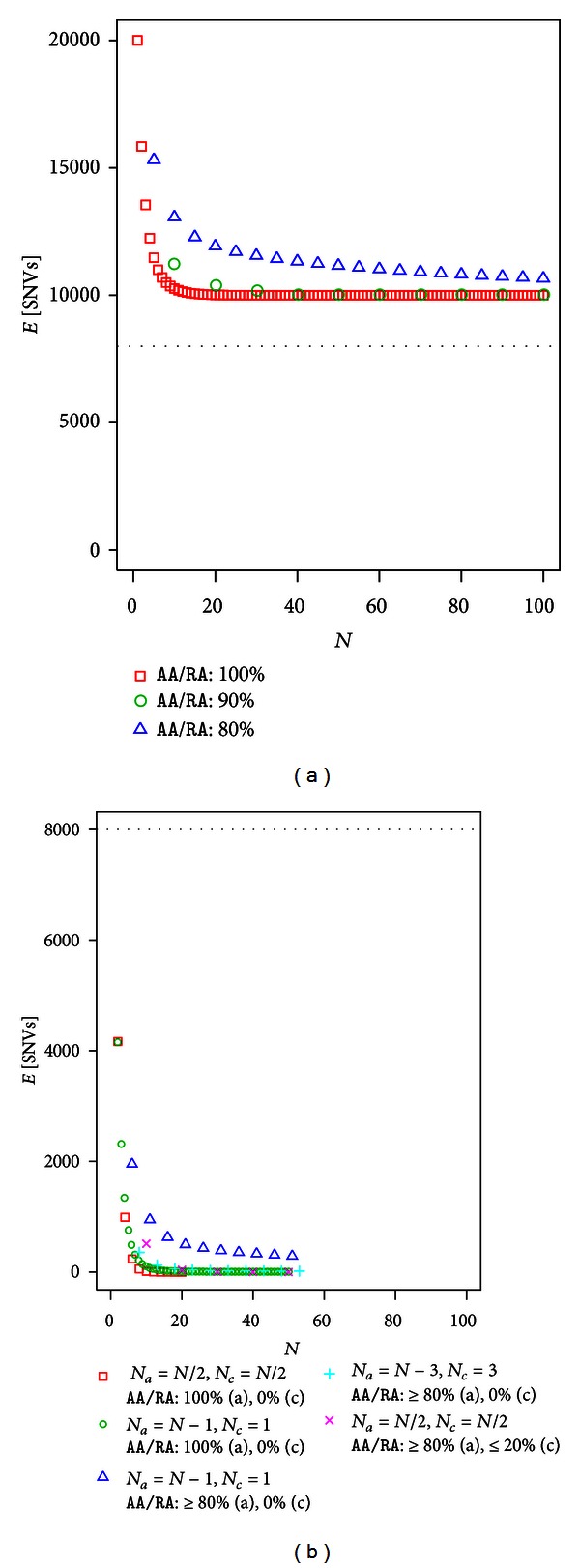
The expected number of SNVs after filtering in dominant disease using full-sibs (a) without control using and (b) with controls.

The expected number of SNVs after filtering in the case of full-sibs for dominant disease is shown in Figure 4.  Table 4 lists several of the values used in Figure 4. Filtering efficacy in sibs was clearly worse than that in unrelated individuals (cf.  Figure 4(a) with Figure 2(a)). For a given sample size *N*, the expected number of SNVs for 100%, 90%, and 80% filtering was relatively similar compared to unrelated individuals. There was also a higher asymptotic value for 100%, 90% and 80% filtering. The asymptotic value was 3*θ*/4 = 9999.75, as explained below based on 100% filtering. When the sample size *N* is large, DNA sites in which the parents have genotypes *RR* × *RA* or *RA* × *RA* are removed by filtering because a certain proportion of sibs have the genotype *AA* (Table 1). In contrast, even if the sample size is large, DNA sites in which the parents have genotypes of *RR* × *AA*, *RA* × *AA*, or *AA* × *AA* are not removed and the expected site is shown as *θ*/6 + *θ*/3 + *θ*/4 = 3*θ*/4 (Table 1). With 90% and 80% filtering, this is correct. 

However, the situation drastically improved when we used controls (Figure 4(b)). For a given sample size *N*, the expected number of SNVs in sibs was comparable to the expected number in unrelated individuals. For example, if half of the exome samples were controls, the expected SNVs in which at least 80% of affected individuals and at most 20% of controls have the genotype *AA*/*RA* were 487.69 (*N* = 10), 28.51 (*N* = 20) and 0.02 (*N* = 50) when unrelated exomes were used and 512.68 (*N* = 10), 40.85 (*N* = 20), and 0.06 (*N* = 50) when full-sibs exomes were used.

**Figure 5:**
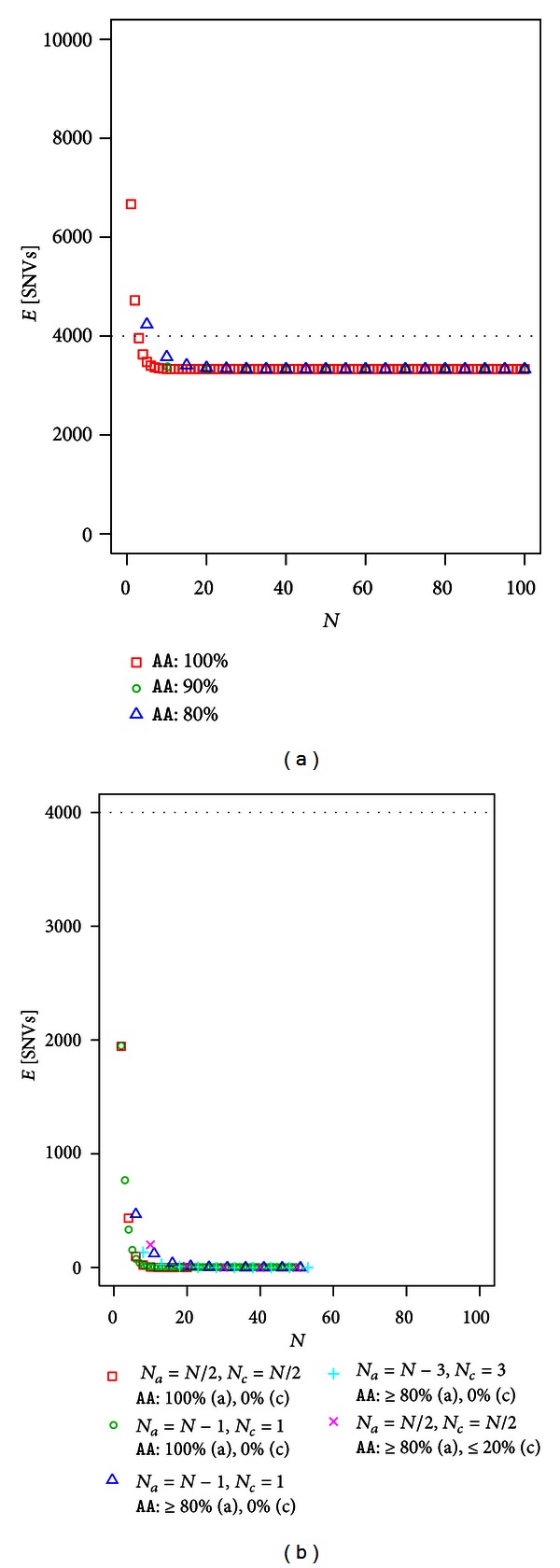
The expected number of SNVs after filtering in recessive disease using full-sibs (a) without control using and (b) with controls.

The number of SNVs after filtering in sibs for recessive disease shows a similar tendency to dominant disease, as shown in Figures 5(a) and 5(b).  Table 5 lists some of the values used in Figure 5. Without controls, the efficiency of filtering in sibs was clearly worse. The asymptotic value for recessive disease was *θ*/4 = 3333.25, which was obtained the same way as for dominant disease. The number of SNVs for recessive disease reached asymptotic values for 100%, 90%, and 80% filtering faster than for dominant disease. The effect of controls in recessive and dominant disease was high. For a given sample size *N*, the expected number of SNVs in sibs was comparable to that in unrelated individuals. For example, if half of the exome samples were controls, the expected SNVs in which at least 80% of affected individuals and at most 20% of controls have the genotype *AA* were 203.70 (*N* = 10), 11.86 (*N* = 20), and 0.01 (*N* = 50) when unrelated exomes were used and 200.09 (*N* = 10), 14.26 (*N* = 20), and 0.02 (*N* = 50) when full-sibs exomes were used.

### 3.6. Assumptions

We assumed that *n* + 1 haploid sequences were randomly sampled from a population under the Wright-Fisher diffusion model with a constant population size, with *n* = 2*N* in *N* unrelated individuals and *n* = 2 in full-sibs (Figures 1(e) and 1(f)). The infinite-site model of neutral mutations was also assumed. The expected frequency spectrum of *n* + 1 sequences is represented by formula (1). All of the results derived from this method are based on this formula. However, human populations have expanded and mutations in non-synonymous sites are not at least strictly neutral but might be averagely deleterious, which may skew the frequency spectrum toward rare variants (e.g., see [9] for population expansion and [10] for non-synonymous mutations). The skew is more pronounced when the sample size is large (e.g., 500), but not when the sample size is small [9]. In addition, the reference sequence is known to be a mosaic of a number of human DNA. The fact does not affect the expected number of candidate SNVs since any small chromosomal region or any DNA site of the reference sequence is still a haploid sample from a population. On the other hand, our results may be affected by the fact that the reference sequence and the exome sequences have different ethnic background. But it is surely that those are derived from a human population. As a whole, the expected frequency spectrum given by (1) is rough approximation and the effect of various filtering manner, incorporating modes of inheritance, incomplete penetrance or phenocopy, and control, on the number of candidate SNVs can be assessed as described above.

## 4. Conclusions and Practical Implications

Using a standard population genetics model, we modeled exome analysis for Mendelian disease and developed a method for calculating the expected number of candidate SNVs after filtering under a “no genetic heterogeneity” assumption. Exome sequences of unrelated individuals and full-sibs were considered with and without controls for dominant and recessive diseases. Without controls, particularly for full-sibs, the filtering approach had poor efficiency in reducing the number of candidate SNVs even when using a stringent filter (Figures 2(a), 3(a), 4(a), and 5(a)). With controls, the filtering efficacy was considerably improved, even when incorporating phenocopy or incomplete penetrance (Figures 2(b), 3(b), 4(b), and 5(b)). This was true in cases of unrelated individuals and full-sibs for dominant and recessive diseases. 

For rare dominant diseases, it is plausible that affected individuals in a pedigree share one disease-causing variant, even if the disease shows genetic heterogeneity. This indicates that the assumption of “no genetic heterogeneity” is appropriate because the frequencies of variants of the rare disease are also rare in a population, and only one founder in the pedigree should have one of the disease-causing variants (e.g., see Sobreira et al. [5] or Wang et al. [11]). For rare recessive diseases, affected members in a pedigree generally do not share one disease-causing variant. It is possible that affected individuals in the pedigree may be “compound heterozygotes” at a disease locus or heterozygotic for two disease-causing variants in a gene (e.g., Lalonde et al. [12]). For a consanguineous pedigree with a rare recessive disease, the assumption of “no genetic heterogeneity” is still appropriate in that affected individuals in the pedigree are expected to be autozygous for the disease-causing variant (e.g., see Walsh et al. [13]).

As described in Section 3.5 and shown in Figure 4(b), filtering by incorporating incomplete penetrance and phenocopy can efficiently reduce the number of candidate SNVs when the sample size is relatively large. If the property of results for full-sibs is extrapolatable to those for general pedigrees, this means that filtering approach works well in case of a pedigree data for dominant disease or a consanguineous pedigree data for recessive disease even in cases of incomplete penetrance and phenocopy. The approach presented in this study could provide general guidelines for sample size determination in exome sequencing for Mendelian disease.

## Figures and Tables

**Table 1:** Expected numbers of SNVs for the parents genotypes and probabilities for sibs genotypes.

Genotype configuration of the parents	Expected number of SNVs	Genotype of sib	Probability for genotype
*RR* × *RA*	*E*[*K*_*RR*×*RA*_]: *θ*	*RR*	1/2
		*RA*	1/2
*RR* × *AA*	*E*[*K*_*RR*×*AA*_]: *θ*/6	*RA*	1
*RA* × *RA*	*E*[*K*_*RA*×*RA*_]: *θ*/3	*RR*	1/4
		*RA*	1/2
		*AA*	1/4
*RA* × *AA*	*E*[*K*_*RA*×*AA*_]: *θ*/3	*RA*	1/2
		*AA*	1/2
*AA* × *AA*	*E*[*K*_*AA*×*AA*_]: *θ*/4	*AA*	1

**Table 2:** The expected number of SNVs after filtering in dominant disease using unrelated individuals.

*N*	*N*_*a*_ = *N*			*N*_*a*_ = *N*/2 *N*_*c*_ = *N*/2	*N*_*a*_ = *N* − 1 *N*_*c*_ = 1	*N*_*a*_ = *N* − 1 *N*_*c*_ = 1	*N*_*a*_ = *N* − 3 *N*_*c*_ = 3	*N*_*a*_ = *N*/2 *N*_*c*_ = *N*/2
	*AA*/*RA*: 100%	*AA*/*RA*: 90%	*AA*/*RA*: 80%	*AA*/*RA*: 100% (*a*) 0% (c)	*AA*/*RA*: 100% (*a*) 0% (c)	*AA*/*RA*: ≥80% (*a*) 0% (c)	*AA*/*RA*: ≥80% (*a*) 0% (c)	*AA*/*RA*: ≥80% (*a*) ≤20% (c)
1	19999.50	—	—	—	—	—	—	—
2	12221.92	—	—	7777.58	7777.58	—	—	—
3	9333.10	—	—	—	2888.82	—	—	—
4	7761.71	—	—	1317.43	1571.39	—	—	—
5	6751.15	—	11803.94	—	1010.56	—	—	—
10	4450.20	7292.89	9899.74	12.68	284.27	—	—	487.69
11	4209.42	—	—	—	240.77	1325.27	—	—
13	3821.65	—	—	—	180.60	—	111.07	—
20	2992.04	6220.39	8915.41	0.01	87.51	—	—	28.51
21	2911.32	—	—	—	80.72	944.79	—	—
23	2767.09	—	—	—	69.48	—	45.77	—
50	1808.56	5540.39	8311.92	—	19.82	—	—	0.02
51	1789.36	—	—	—	—	736.85	—	—
53	1752.68	—	—	—	—	—	21.74	—
100	1249.75	5306.36	8108.38	—	—	—	—	—

**Table 3:** The expected number of SNVs after filtering in recessive disease using unrelated individuals.

*N*	*N*_*a*_ = *N*			*N*_*a*_ = *N*/2 *N*_*c*_ = *N*/2	*N*_*a*_ = *N* − 1 *N*_*c*_ = 1	*N*_*a*_ = *N* − 1 *N*_*c*_ = 1	*N*_*a*_ = *N* − 3 *N*_*c*_ = 3	*N*_*a*_ = *N*/2 *N*_*c*_ = *N*/2
	*AA*: 100%	*AA*: 90%	*AA*: 80%	*AA*: 100% (*a*) 0% (c)	*AA*: 100% (*a*) 0% (c)	*AA*: ≥80% (*a*) 0% (c)	*AA*: ≥80% (*a*) 0% (c)	*AA*: ≥80% (*a*) ≤20% (c)
1	6666.50	—	—	—	—	—	—	—
2	3333.25	—	—	3333.25	3333.25	—	—	—
3	2222.17	—	—	—	1111.08	—	—	—
4	1666.63	—	—	555.54	555.54	—	—	—
5	1333.30	—	2999.93	—	333.33	—	—	—
10	666.65	1407.37	2240.68	5.29	74.07	—	—	203.70
11	606.05	—	—	—	60.60	422.55	—	—
13	512.81	—	—	—	42.73	—	41.83	—
20	333.33	1054.55	1863.36	0.00	17.54	—	—	11.86
21	317.45	—	—	—	15.87	276.10	—	—
23	289.85	—	—	—	13.17	—	15.73	—
50	133.33	843.18	1637.71	—	2.72	—	—	0.01
51	130.72	—	—	—	—	199.84	—	—
53	125.78	—	—	—	—	—	6.80	—
100	66.67	772.77	1562.62	—	—	—	—	—

**Table 4:** The expected number of SNVs after filtering in dominant disease using full-sibs.

*N*	*N*_*a*_ = *N*			*N*_*a*_ = *N*/2 *N*_*c*_ = *N*/2	*N*_*a*_ = *N* − 1 *N*_*c*_ = 1	*N*_*a*_ = *N* − 1 *N*_*c*_ = 1	*N*_*a*_ = *N* − 3 *N*_*c*_ = 3	*N*_*a*_ = *N*/2 *N*_*c*_ = *N*/2
	*AA*/*RA*: 100%	*AA*/*RA*: 90%	*AA*/*RA*: 80%	*AA*/*RA*: 100% (*a*) 0% (c)	*AA*/*RA*: 100% (*a*) 0% (c)	*AA*/*RA*: ≥80% (*a*) 0% (c)	*AA*/*RA*: ≥80% (*a*) 0% (c)	*AA*/*RA*: ≥80% (*a*) ≤20% (c)
1	19999.50	—	—	—	—	—	—	—
2	15832.94	—	—	4166.56	4166.56	—	—	—
3	13541.33	—	—	—	2291.61	—	—	—
4	12239.28	—	—	989.56	1302.05	—	—	—
5	11471.07	—	15312.12	—	768.21	—	—	—
10	10263.05	11227.51	13064.81	14.05	96.45	—	—	512.68
11	10193.97	—	—	—	69.08	948.55	—	—
13	10106.96	—	—	—	36.82	—	127.64	—
20	10013.86	10408.02	11922.23	0.01	4.71	—	—	40.85
21	10010.33	—	—	—	3.53	500.32	—	—
23	10005.70	—	—	—	1.98	—	38.66	—
50	9999.75	10031.07	11165.22	—	0.00	—	—	0.06
51	9999.75	—	—	—	—	291.41	—	—
53	9999.75	—	—	—	—	—	18.23	—
100	9999.75	10000.36	10661.20	—	—	—	—	—

**Table 5:** The expected number of SNVs after filtering in recessive disease using full-sibs.

*N*	*N*_*a*_ = *N*			*N*_*a*_ = *N*/2 *N*_*c*_ = *N*/2	*N*_*a*_ = *N* − 1 *N*_*c*_ = 1	*N*_*a*_ = *N* − 1 *N*_*c*_ = 1	*N*_*a*_ = *N* − 3 *N*_*c*_ = 3	*N*_*a*_ = *N*/2 *N*_*c*_ = *N*/2
	*AA*: 100%	*AA*: 90%	*AA*: 80%	*AA*: 100% (*a*) 0% (c)	*AA*: 100% (*a*) 0% (c)	*AA*: ≥80% (*a*) 0% (c)	*AA*: ≥80% (*a*) 0% (c)	*AA*: ≥80% (*a*) ≤20% (c)
1	6666.50	—	—	—	—	—	—	—
2	4722.10	—	—	1944.40	1944.40	—	—	—
3	3958.23	—	—	—	763.87	—	—	—
4	3628.38	—	—	434.02	329.85	—	—	—
5	3476.48	—	4236.01	—	151.91	—	—	—
10	3337.59	3381.12	3578.15	5.37	4.35	—	—	200.19
11	3335.42	—	—	—	2.17	122.91	—	—
13	3333.79	—	—	—	0.54	—	31.16	—
20	3333.25	3334.14	3359.51	0.00	0.00	—	—	14.26
21	3333.25	—	—	—	0.00	13.13	—	—
23	3333.25	—	—	—	0.00	—	3.28	—
50	3333.25	3333.25	3333.30	—	0.00	—	—	0.02
51	3333.25	—	—	—	—	0.03	—	—
53	3333.25	—	—	—	—	—	0.01	—
100	3333.25	3333.25	3333.25	—	—	—	—	—
